# Cinnamaldehyde Promotes the Intestinal Barrier Functions and Reshapes Gut Microbiome in Early Weaned Rats

**DOI:** 10.3389/fnut.2021.748503

**Published:** 2021-10-12

**Authors:** Lili Qi, Haiguang Mao, Xiaohui Lu, Tingting Shi, Jinbo Wang

**Affiliations:** ^1^School of Biological and Chemical Engineering, NingboTech University, Ningbo, China; ^2^Ningbo Biomart Lifetech Co. Ltd, Ningbo, China

**Keywords:** cinnamaldehyde, gut barrier, inflammatory responses, gut microbiota, early weaned rats

## Abstract

Cinnamaldehyde is an aromatic aldehyde isolated from the essential oil of cinnamon. It has been proved to possess various bioactivities such as anti-inflammation, anti-bacteria and antihypertensive. Nevertheless, early weaning could lead to intestinal stress, causing a range of intestinal health problems. The aim of this study is to explore the effects of cinnamaldehyde on gut barrier integrity, inflammatory responses, and intestinal microbiome of early weaned rats. In this study, treatment with cinnamaldehyde (100 or 200 mg/kg bodyweight/day) for 2 weeks significantly promoted the production of mucins in the colonic epithelial tissue of rats. Cinnamaldehyde supplementation significantly upregulated the expression of Muc2, TFF3 and the tight junction proteins (ZO-1, claudin-1, and occludin). Hematoxylin and eosin staining results showed that colonic histopathological changes were recovered by cinnamaldehyde supplementation. The mRNA expression of IL-6 and TNF-α were significantly decreased in the cinnamaldehyde groups while the TNF-α protein levels were significantly decreased in the two cinnamaldehyde groups. Cinnamaldehyde treatment obviously attenuated the activation of NF-κB signaling pathway in rat colonic tissue and suppressed the production of inflammatory cytokines. Furthermore, cinnamaldehyde supplementation remodeled the gut microbiome structure, at the genus level, *Akkermansia, Bacteroides, Clostridium III, Psychrobacter, Intestinimonas* were increased, whereas those of *Ruminococcus, Escherichia/Shigella* were obviously decreased in the cinnamaldehyde treated groups. These findings indicated that cinnamaldehyde could effectively enhance intestinal barrier integrity, ameliorate inflammatory responses and remodel gut microbiome in early weaned rats.

## Introduction

Cinnamaldehyde is the major bioactive component isolated from cinnamon essential oils. Previous studies have shown that cinnamaldehyde exhibits a wide range of biological activities including anti-inflammatory, anti-bacterial, and immune-modulating properties (1–3). As the major component of cinnamon, cinnamaldehyde has been traditionally used as a food additive, and registered as a flavoring agent by the Food and Drug Administration and approved for food use (4).

Gut is an important barrier which protects against the entry of pathogenic bacteria and harmful macromolecules into the body. The immature barrier function plays key roles in the pathogenesis of intestinal inflammatory diseases of newborns and children, such as inflammatory bowel disease (IBD), infectious enteritis, or necrotizing enterocolitis (5). Previous study has suggested that the infant maturation of the intestinal epithelium has lifelong impacts on gut functions and immune homeostasis (6). The suckling-to-weaning dietary transition influences the maturation of gut barrier in mammals. The transition from maternal milk to solid food also results in the remodeling of gut microbiota composition. The intestinal bacterial metabolites were found to be the key intermediates which induced the maturation of gut and formation of intestinal barrier (7).

Many natural bioactive compounds have been found to promote intestinal maturation and modulate the gut microbiota composition (8, 9). Previous investigations suggested that cinnamaldehyde inhibited PLCγ-1 activation in mucosal mast cells, attenuated the inflammatory responses and ameliorated ulcerative colitis in the rat models (10, 11). To our knowledge, the effects of cinnamaldehyde on the gut integrity and mucosal immune functions in juvenile animals have not been explored. Weaning, especially early weaning, is a stressful condition for mammals and negatively affects growth performance by affecting the development of mucosal barrier function in the intestine (12). Early weaning (15- to 21-day weaning age) resulted in sustained impairment in intestinal barrier function, and commonly results in gastrointestinal disorders, inflammation and diarrhea in infants and young animals (13). Therefore, in the current study, we used early weaned rats as the animal model to investigate the effects of cinnamaldehyde on the intestinal barrier, mucosal immune functions and gut microbiota profile in the early weaned rats.

## Materials and Methods

### Materials and Reagents

Cinnamaldehyde was purchased from Sigma-Aldrich (St.Louis, MO, USA). The total RNA Kit and Stool DNA Kit were purchased from OMEGA Bio-Tek (Norcross, GA, USA). One-step qRT-PCR kits were purchased from TOYOBO (Osaka, Japan). Anti-NF-κB p65, anti-phospho-NF-κB p65, anti-TNF, anti-IL-6 and HRP-linked goat anti-rabbit antibody were obtained from Cell Signaling Technology (Beverly, MA, USA). ECL Western Blotting Substrate was supplied by Thermo Fisher (Shanghai, China). RIPA lysis buffer and BCA protein assay kits were obtained from Beyotime (Shanghai, China). Nitrocellulose membranes were purchased from Sigma-Aldrich (St.Louis, MO, USA). AB/PAS staining kits were purchased from Solarbio Life Science (Beijing, China). Enzyme-linked immunosorbent assay (ELISA) kits for estimating TNF-α, and IL-6 levels were obtained from Multisciences Biotech (Hangzhou, China).

### Animal Experiment Design

Twenty four early weaned male SPF SD rats (17 days) were purchased from the Laboratory Animal Center of Zhejiang Province (Hangzhou, China). The rats had free access to water and were fed in a temperature-controlled room (23–25°C) under a 12-h dark-light cycle. The experimental procedure was approved by the ethical committee in Zhejiang University, and was decided following the rule of the NIH Guide for the Care and Use of Laboratory Animals (NIH Publication No. 85-23, 1985, revised 1996). The immature rats were removed from their dams at 17 days of age (weaned), following 3 days of adaptive feeding with the diet of rat formula feeds mixed with corn, soybean meal, fish meal, flour, yeast powder, vegetable oil, salt, a variety of vitamins, and mineral elements. The diet contains 18% protein, 4% fat, 5% crude fiber, 1.8% Ca, and 1.2% phosphorus with the energy content of 3.4 kcal/g. The rats were weighed after 3 days (21 days of age) of adaptive feeding, then they were randomly divided into 3 groups with 8 rats per group: control, CIN100 and CIN200. In addition, each mouse was raised separately. The control group was feed with the above formula feeds, and the CIN100 and CIN200 groups were fed with the above formula feeds mixed with the fresh liquid cinnamaldehyde of 100 or 200 mg/kg body weight/d, respectively (the liquid cinnamaldehyde was added directly into formula feeds). The reason why cinnamaldehyde was administered as the amounts of 100–200 mg/kg was the result of the previous pre-test and reference of the previous research reports (14). After 7 days of feeding experiment (28 days of age), euthanasia was carried out by intraperitoneal injection of pentobarbital sodium of 150 mg/kg body weight until the animal stopped breathing, then the abdomen was incised to obtain the colon tissue samples.

### Alcian Blue-Periodic Acid Schiff (AB/PAS) Staining

Alcian blue-periodic acid schiff (AB/PAS) staining was conducted to observe the variation of goblet cells. Five millimeter of colonic tissue was immediately fixed in Carnoy's fluid at 4°C for 2 h. Fixed colon tissues were embedded in paraffin and cut into 5 μm sections and subjected to AB/PAS staining. The variation of goblet cells and integrity of mucus were analyzed using a microscope (Nikon E100, Tokyo, Japan). ImageJ software was used for data acquisition and image analysis.

### Hematoxylin and Eosin (H&E) Staining

The colonic tissues were fixed in 10% neutral buffered formalin and then transferred to 70% ethanol. Fixed tissues were embedded in paraffin and cut into 4 μm thick slices. Tissues were stained with hematoxylin and eosin (H&E). The histological changes were observed with optical microscopy (Nikon, Tokyo, Japan). Sections were evaluated based on the cell infiltration of inflammatory cells and epithelial damage as previously described.

### RT-qPCR

Total RNA was extracted from colonic tissues using the total RNA Kit (OMEGA Bio-Tek). RNA concentrations were determined at 260 nm and purity was assessed by the A260/A280 nm ratio. RT-qPCR was performed using CFX Connect System (Bio-Rad, California, USA) with one-step RT-qPCR Kit (TOYOBO) according to the manufacturer's protocols. The sequences of the primers were listed in Table 1. Thermal cycling conditions were as follows: 2 min denaturation at 98°C, followed by 40 cycles at 98°C at 10 s, 10 s at 60°C, 30 s at 68°C. Data were collected and analyzed using the CFX Manager software (Bio-Rad, California, USA). Cycle thresholds were normalized to GAPDH levels and fold changes were calculated to the normalized control of each gene. The relative mRNA levels were examined using the ΔΔCt method. Each sample was treated in triplicate to ensure statistical analysis significance.

**Table 1:** Primers used for qRT-PCR.

	**Forward primer (5′-3′)**	**Reverse primer (5′-3′)**
MUC2	GCTGACGAGTGGTTGGT GAATG	GATGAGGTGGCAGACAGGAGAC
TFF3	CCGTGGTTGCTGTTTTGAC	GCCTGGACAGCTTCAAAATG
ZO-1	ACCCGAAACTGATGCTGTG GATAG	AAATGGCCGGGCAGAACTTGTGTA
claudin-1	AGCTGCCTGTTCCATGTACT	CTCCCATTTGTCTGCTGCTC
occludin	ACGGACCCTGACCACTATGA	TCAGCAGCAGCCATGTACTC
TNF-α	CCCTCACACTCAGATCAT CTTCT	CTACGACGTGGGCTACAG
IL-6	CTCTGGCGGAGCTATTGAGA	AAGTCTCCTGCGTGGAGAAA
GAPDH	GAAGGTGAAGGTCGGAG TCAAC	CATCGCCCCACTTGATTTTGGA

### Western Blot Analysis

The fresh colon tissue was washed for three times with pre-cooling PBS of 4°C. Filter paper was used to absorb the rest liquid on the tissue surface and then cut the colon tissue into several smaller tissue pieces. Add the tissue pieces into RIPA (Radio Immunoprecipitation Assay) buffer (Beyotime, Shanghai, China) in a ratio of tissue weight (g): lysate (mL) = 1:10, and homogenized using a homogenizer until no obvious tissue mass could be observed, then incubated on ice for 30 min. Centrifuged 10,000 g at 4°C for 10 min, the supernatant was the extracted protein. Fifty microgram of protein samples were separated by either 3–8% Tris-acetate gradient gels for MUC2 detection or 12.5% Tris-glycine gels for detection of other proteins. Then the protein samples were transferred to nitrocellulose membranes (Sigma-Aldrich, St. Louis, MO, USA). The membranes were blocked with 5% skim milk in TBS-T for 2 h and then washed with TBS-T for three times at 4°C. The blocked membranes were incubated in primary antibody at 4°C overnight, followed by incubation with secondary antibody at room temperature for 1 h. ECL Substrate (Thermo Fisher, Shanghai, China) was used to image the protein bands with Chemi Doc XRS system (Bio-Rad, CA, USA). The optical density was analyzed by Quantity one 4.6.2 software. The antibodies used were shown as follows: anti-Claudin-1 (Invitrogen, Cat.37-4900), anti-Occludin (Invitrogen, Cat.33-1500), anti-ZO-1 (Invitrogen, Cat.61-7300), anti-P-NFκB (CST, Cat.3003), anti-NF-κB (CST, Cat.8242), anti-TNF alpha (Abcam, Cat.ab183218), anti-IL6 (Abcam, Cat.ab259341), and anti-β-actin (Abcam, Cat.ab8226).

### Enzyme-Linked Immunosorbent Assay (ELISA)

To determine the protein levels of TNF-α and IL-6, the colon tissue was homogenized with cold 10 mM PBS (pH 7.4, containing 1 mM phenylmethylsulfonyl fluoride). After that, the mixture was centrifuged at 10,000×g for 5 min at 4°C. The supernatants were collected and detected by using TNF-α or IL-6 ELISA kits (Multisciences Biotech, Hangzhou, China) according to the manufacturer's instructions. The TNF-α and IL-6 levels were expressed as picograms per gram of tissue protein.

### Analysis of Gut Microbiota

The structural changes of gut microbiome were conducted by 16S rRNA sequencing analysis. Total DNA was extracted with the E.Z.N.A. Stool DNA Kit (Omega, Norcross, GA, USA) according to instructions. The collection of feces was carried out in a sterile environment in a super clean table. Fixed the experiment rat and lifted its tail, gently pressed the abdomen with fingers to collect fresh feces and put them into a sterile EP tube, each tube contained 2–3 fecal particles. The bacterial hypervariable V3–V4 region of 16S rRNA was amplified by using primer: 341F: CCCTACACGACGCTCTTCCGATCTG and 805R: GACTGG+AGTTCCTTGGCACCCGAGAATTCCA. The validated library was used for sequencing on HiSeq 2500 (Illumina, CA, USA). The high quality paired-end reads were combined to tags based on overlaps by FLASH (Fast Length Adjustment of Short reads, v1.2.11), and then clustered into Operational Taxonomic Units (OTUs) at a similarity cutoff value of 97% using USEARCH (v7.0.1090), and chimeric sequences were compared with Gold database using UCHIME (v4.2.40) to detect. Alpha diversities (Shannon and Simpson) and richness (ACE and Chao1) were obtained using mother (version 1.30.1). Beta diversity was determined using OTUs from each sample. Gut microbiota compositions of the groups at different levels (phylum and genus) were analyzed using MUSCLE software (version 3.8.31). Mothur software and Metastats statistical algorithms were used to compare the relative bacterial abundances at the phylum and genus levels, and significant differences in taxonomic compositions were analyzed via the Kruskal Wallis test. The data was uploaded to SAR with the accession NO. PRJNA761503.

### Statistical Analysis

SPSS20.0 (SPSS, Chicago, IL) was used for statistical analysis, a one-way analysis of variance (ANOVA) was carried out for each comparison, followed by *post hoc* analysis to identify differences between specific factor levels using the Tukey's Honest Significant Difference (HSD) test. *P* < 0.05 were regarded as statistically significant.

## Results and Discussion

### Cinnamaldehyde Promotes the Production of Colonic Mucin

**Figure 1:**
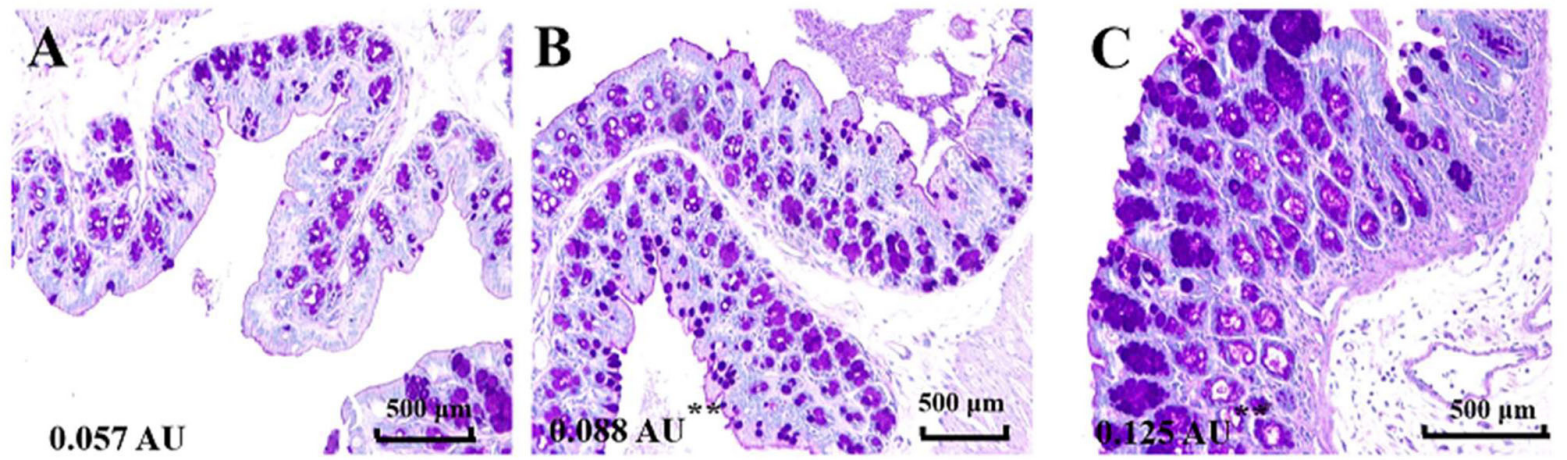
Cinnamaldehyde treatment promoted the production of mucins. The variation of goblet cells in different treatment groups was analyzed by AB/PAS staining (200×, *n* = 3). **(A)** Control. **(B)** CIN100. **(C)** CIN200. The mean fluorescence intensity of each picture is 0.057, 0.088**, 0.125** arbitrary units (AU) using ImageJ software. One-way ANOVA was carried out for each comparison with HSD test.

Mucus is the first mechanical defense in the intestinal barrier, which protects against the invasion of intestinal bacteria and entry of harmful macromolecules (15). Damage of mucus integrity would result in leaky gut, promote the translocation of bacteria and induce intestinal inflammatory responses (16). To investigate the influence of cinnamaldehyde on the intestinal mucus, we visualized the colonic epithelial layer by staining with AB/PAS after 7 days of treatment with cinnamaldehyde (Figure 1). The results showed that treatment with cinnamaldehyde significantly (*P* < 0.01) increased the amount of secretary granules in the colonic epithelial tissues compared to the control (Figure 1). This study suggested that cinnamaldehyde might enhance the host defense via promoting the production of mucins.

### Cinnamaldehyde Supplementation Improved the Gut Barrier Integrity in Newly Weaned Rats

**Figure 2:**
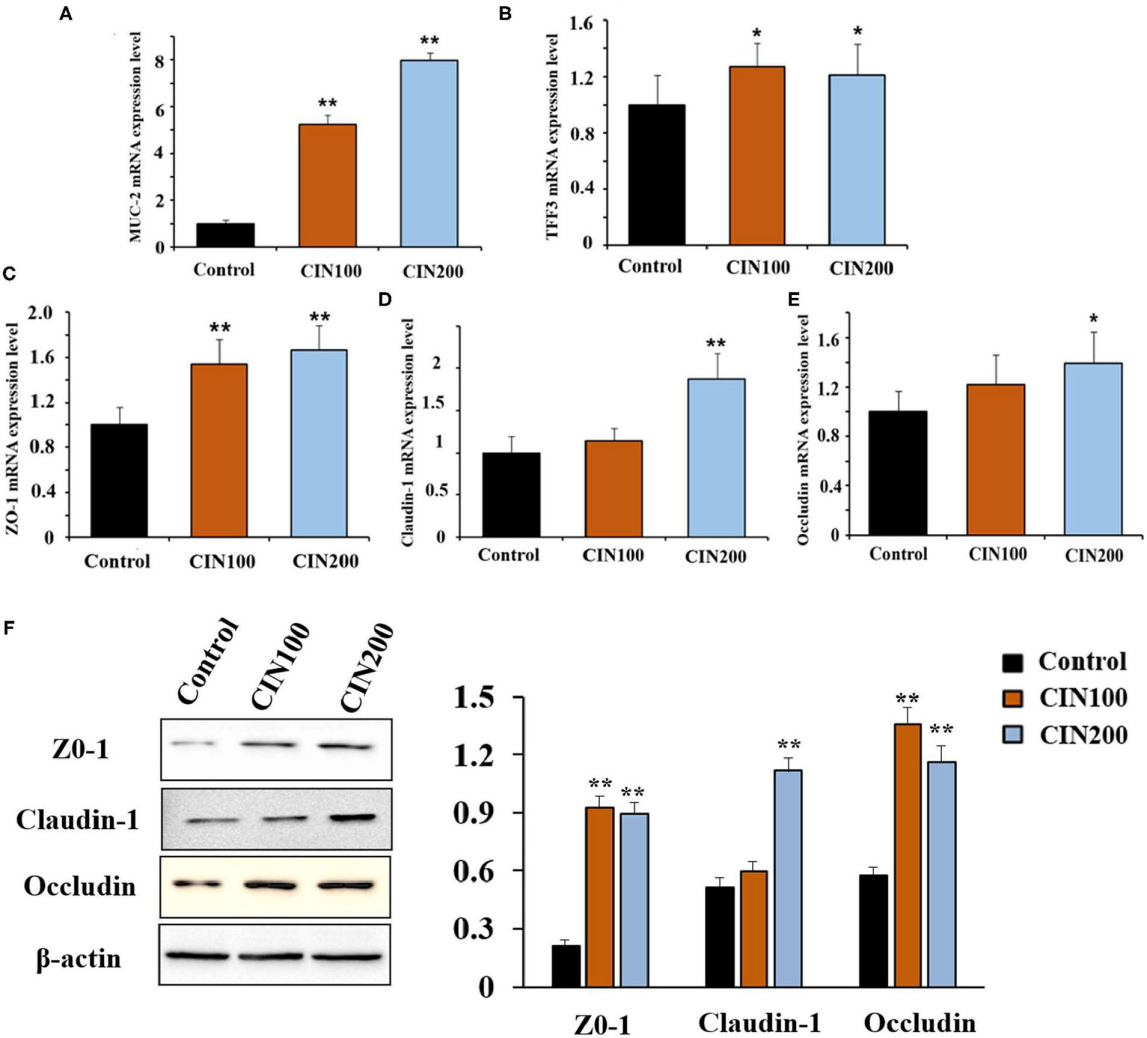
Cinnamaldehyde supplementation promotes the mRNA levels of MUC2, TFF3, TJs, and protein levels of TJs (ZO-1, Claudin-1, and Occludin) in early weaned rats. **(A)** MUC2. **(B)** TFF3. **(C)** ZO-1. **(D)** Claudin-1. **(E)** Occludin. **(F)** Protein levels of TJs. mRNA levels were normalized by GAPDH and protein levels were normalized β-actin. Data are expressed as mean ± SD. *P < 0.05, **P < 0.01. One-way ANOVA was carried out for each comparison with HSD test.

Increased permeability is thought to be associated with intestinal pathogenesis (17, 18). The intestinal mucus in juvenile animals is immature, which is easy to be infected by intestinal bacteria. In the colon, MUC2 is a predominant secretory mucin expressed in goblet cells (19). Trefoil factor 3 (TFF3) is an important mucosal protective factor which usually enhances the protective properties of the mucus layer in a cooperative manner with MUC2 (20). It has been reported that the expression of MUC2 and TFF3 could be stimulated by phytonutrient such as eugenol, carvacrol, and cinnamaldehyde (21). Here we found that cinnamaldehyde treatment markedly enhanced the mRNA expression of MUC2 and TFF3 in the colon compared with control group (Figures 2A,B).

Tight junctions (TJs) also play important roles in the maintenance of intestinal permeability, which is considered to determine selective para-cellular absorption (18). Previous studies have shown that the zonula occludins (ZO), claudin family proteins, and occludin are essential components of TJs in the epithelial barrier (22). The major functions of TJs are to maintain the integrity of the intestinal epithelial barrier. RT-qPCR results showed that treatment with cinnamaldehyde significantly up-regulated the mRNA expression of TJs such as ZO-1 (Figure 2C), claudin-1 (Figure 2D), and occluding (Figure 2E). In the CIN100 group, the mRNA levels of ZO-1, claudin-1, and occludin were elevated by 54, 14, and 22%, respectively, compared with the control group. In the CIN200 group, the mRNA levels of ZO-1, claudin-1, and occludin were elevated by 67, 87, and 39%, respectively. Moreover, WB results also showed that treatment with cinnamaldehyde significantly up-regulated the protein expression levels of TJs such as ZO-1, claudin-1, and occluding (Figure 2F). Consistent with these results, a few other phytochemicals, such as flavonoids and polyphenols, have also been indicated to enhance TJ functions and gut integrities (22–24).

### Cinnamaldehyde Attenuates the Inflammatory Responses in the Colon Tissues

**Figure 3:**
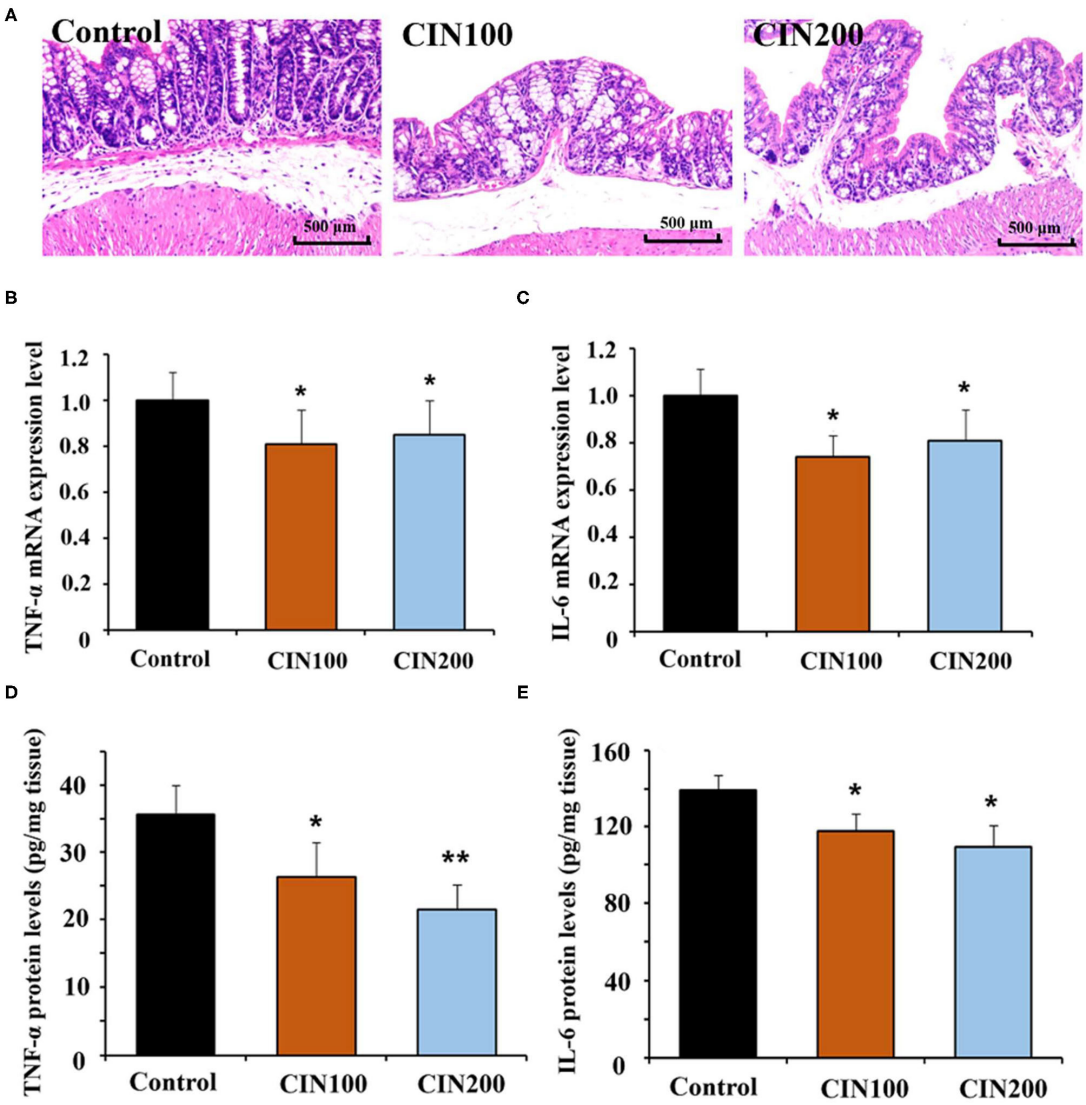
Cinnamaldehyde supplementation attenuates the inflammatory responses in early weaned rat colon. **(A)** H&E stained distal colon sections from untreated and cinnamaldehyde treated rats at day 7 (Original magnification 100×). Inflammation is evident in control group rats. Quantitative RT-PCR was performed to detect the mRNA expression of TNF-α **(B)** and IL-6 **(C)**, normalized by GAPDH. ELISA was conducted to measure the production of TNF-α **(D)** and IL-6 **(E)** in rat colonic tissues. Data are represented as mean ± SD. *P < 0.05, **P < 0.01. One-way ANOVA was carried out for each comparison with HSD test.

It has been reported that cinnamaldehyde could play anti-inflammatory activities in infection, injury and autoimmune disease (14, 25). Histological analysis showed clear inflammation in the colon of newly weaned rats, while treatment with cinnamaldehyde markedly ameliorated the histological changes (Figure 3A). These results suggested that cinnamaldehyde treatment could attenuate the colonic inflammatory responses of early weaned rats. As cytokines play key roles in the inflammatory responses, the expression and production of cytokines (TNF-α and IL-6) have been determined. The results showed the mRNA expression of IL-6 and TNF-α were significantly decreased in the cinnamaldehyde groups (Figures 3B,C). Compared with the control group, TNF-α protein levels were significantly decreased in the two cinnamaldehyde groups. In the control group, the concentration of IL-6 was 139.4 ± 7.7 pg/g tissue, while the levels of IL-6 in CIN100 and CIN200 were 117.7 ± 9.3 pg/g tissue and 109.4 ± 11.2 pg/g tissue, respectively (Figures 3D,E). Our results demonstrate cinnamaldehyde could attenuate the intestinal inflammatory responses in newly weaned rats. These results are in line with the previous study which reported that cinnamaldehyde suppressed the production of TNF-α and IL-6 in ulcerative colitis model mice (11).

### Cinnamaldehyde Inhibited the Activation of NF-κB Signaling Pathway in the Colon Tissues

**Figure 4:**
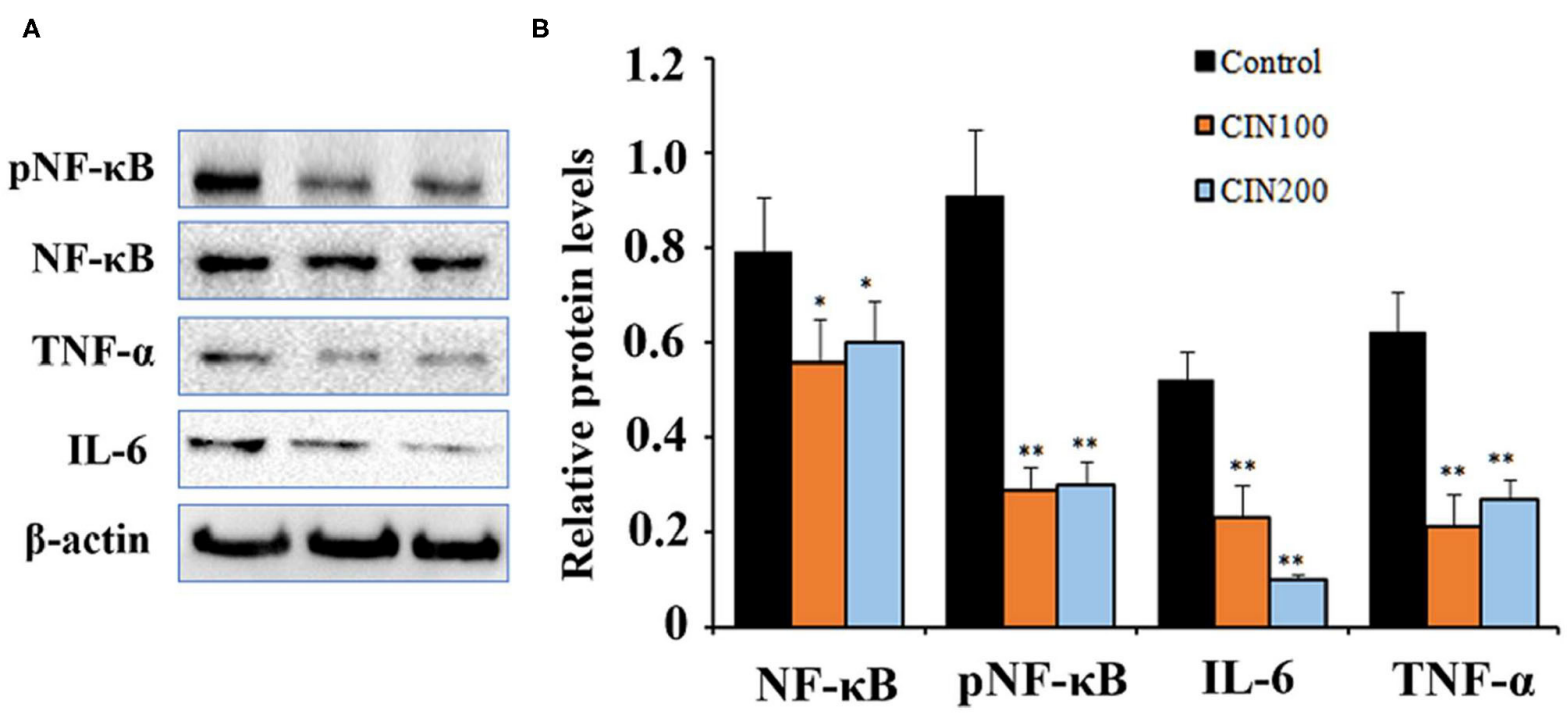
Cinnamaldehyde supplementation inhibits the activation of NF-κB in early weaned rat colon tissues. **(A)** Western blot analysis for NF-κB, pNF-κB and the downstreaming inflammatory cytokines. **(B)** Quantitative analysis for the densitometry of the proteins performed using ImageJ software. Data are expressed as mean ± SD. *P < 0.05, **P < 0.01. One-way ANOVA was carried out for each comparison with HSD test.

NF-κB signaling pathway play important roles in the inflammatory responses (26). NF-κB exists as an inactive form in the cytoplasm, which is a p50/p65 heterodimer and associated with the inhibitor of nuclear factor-kappa B (IκB). After being stimulated by external stimuli, the p65 is phosphorylated by the protein kinases and then the phosphorylated NF-κB translocates to the nucleus and promotes the expression of downstream inflammatory factors (27). When the signaling pathway is activated, the ratio of pNF-κB/NF-κB is markedly elevated (28). To explore the effects of cinnamaldehyde on NF-κB signaling pathway in colonic tissues of newly weaned rats, western blot analysis was performed to determine the levels of the key proteins. The results showed that when the rats were administrated with 100 or 200 mg cinnamaldehyde/kg body weight, the level of colonic pNF-κB was reduced 54 and 51% (Figure 4A), respectively. Compared to the control group, the ratios of pNF-κB/NF-κB were also markedly decreased. The expression levels of TNF-α showed 91% reduction in both cinnamaldehyde groups, while the levels of IL-6 were reduced 44 and 88% (Figure 4B), respectively. These results suggested that cinnamaldehyde treatment could attenuate the inflammatory responses through inhibiting the activation of NF-κB signaling pathway. Our results were supported by a previous report that cinnamaldehyde could inhibit the NF-κB activation and attenuate inflammatory responses (16).

### Cinnamaldehyde Altered Microbial Composition in Colon of Early Weaned Rats

**Figure 5:**
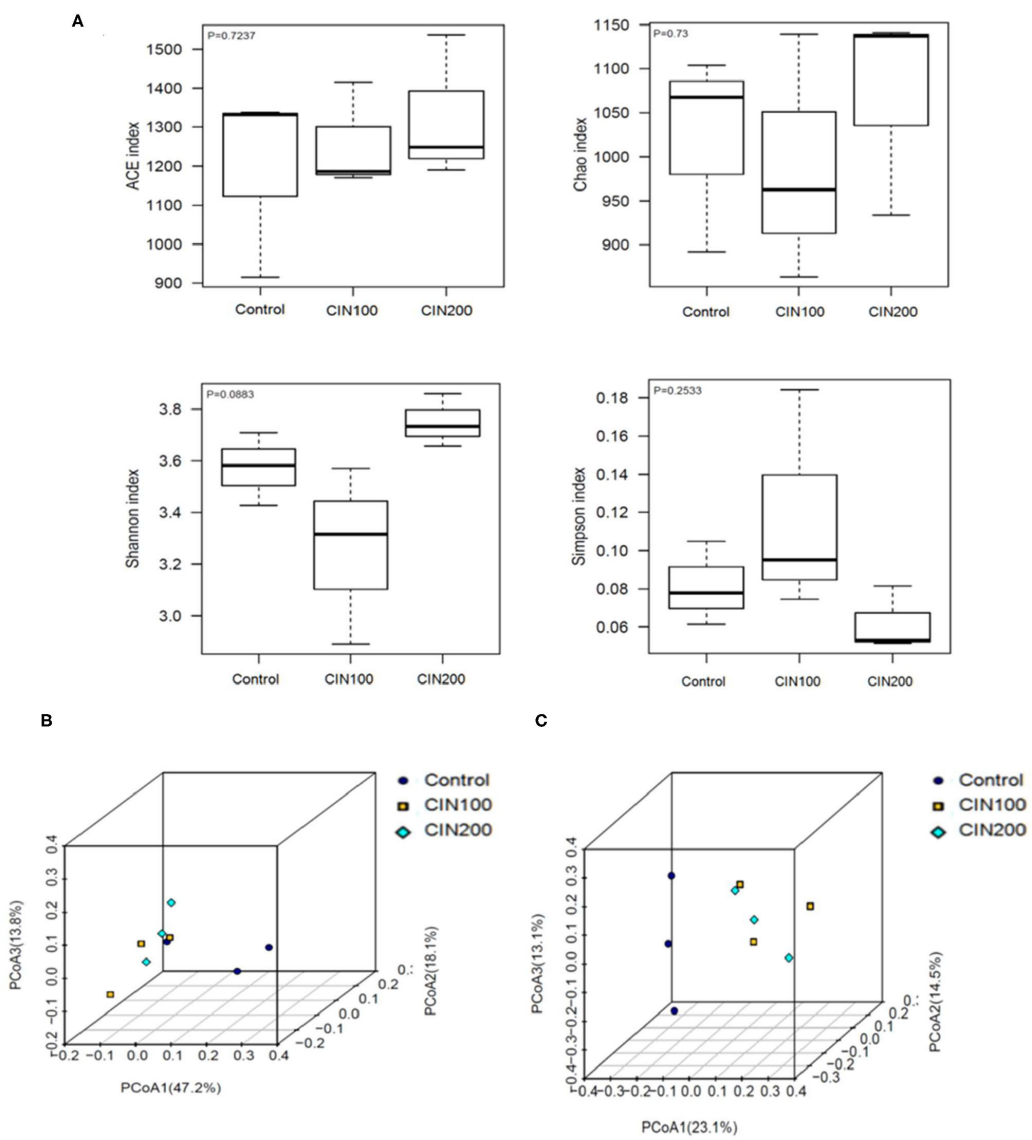
The effects of Cinnamaldehyde supplementation on the α- and β-diversity of gut microbiota communities. **(A)** Alpha diversity metrics for the gut microbiota treated with cinnamaldehyde. **(B)** Beta diversity presented as weighted UniFrac distances. **(C)** Beta diversity presented as unweighted UniFrac distances. One-way ANOVA was carried out for each comparison with HSD test.

The impact of cinnamaldehyde on alpha diversity of the gut microbiota community was determined based on ACE Diversity Index, Chao Diversity Index and Shannon's Diversity Index (Figure 5A). These measures of diversity were calculated following the creation of a rooted phylogenetic tree using OTUs generated from 16s rRNA sequencing. Species Richness is a measure of the number of different species in each sample. As shown in Figure 5A, there was no significant difference between the control and cinnamaldehyde treated groups, a measure of the diversities of the species in each sample exhibited no significant differences between any of the experimental groups. These results showed that cinnamaldehyde couldn't cause a consistent disruption in the alpha diversity of the human gut microbial community. The impact of cinnamaldehyde on the beta diversity of the human gut microbial community was determined using weighted and unweighted UniFrac distance PCoA analysis. The principal coordinates analysis (PCoA) based on the Weighted and Unweighted UniFrac algorithm clearly revealed gut microbial community altered between cinnamaldehyde groups and control group (Figures 5B,C). There was no significant difference between the two cinnamaldehyde groups. This suggested the majority members of the microbial community didn't differ dramatically in cinnamaldehyde groups, whereas the composition of the microbial community was distinct from control group.

**Figure 6:**
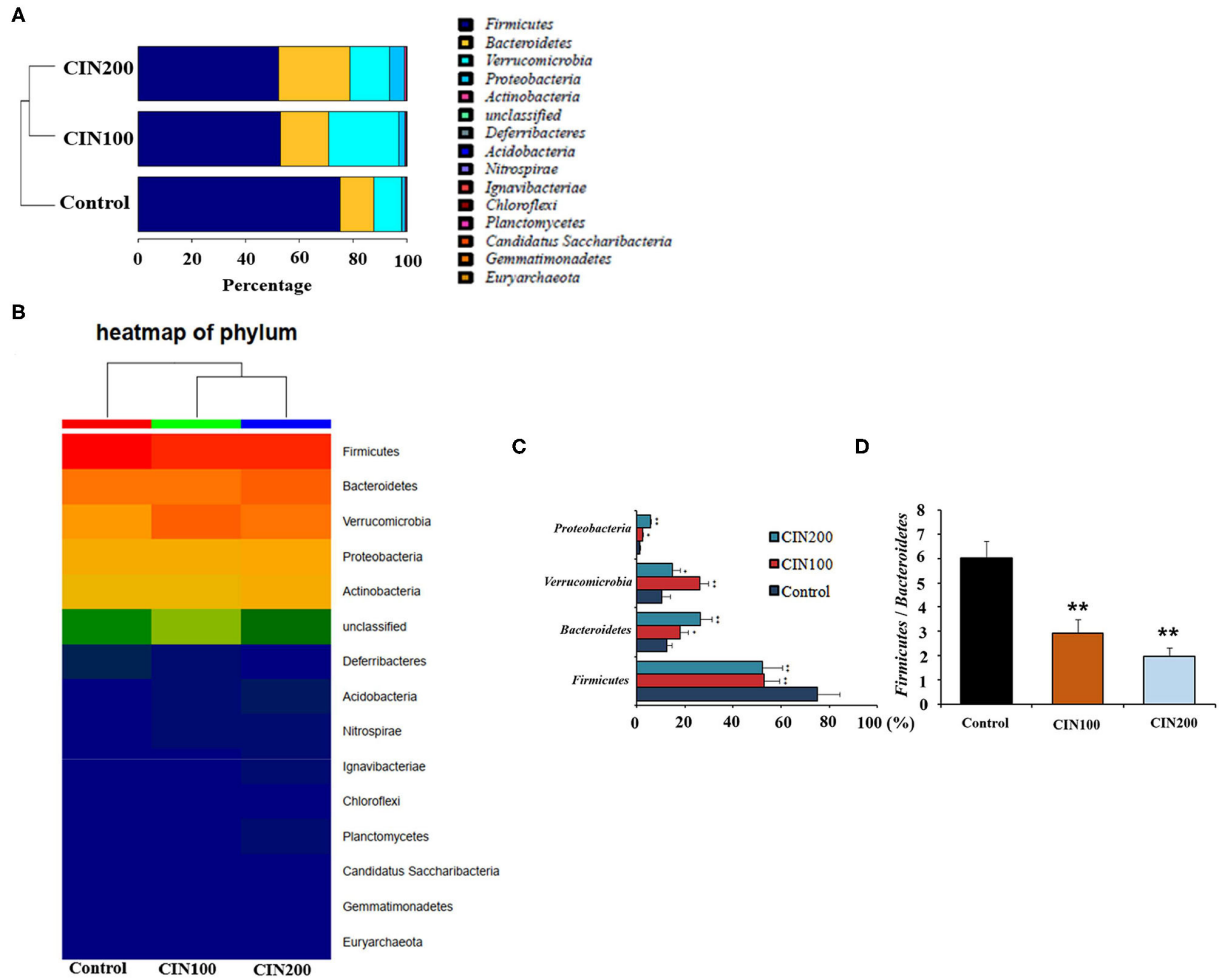
Cinnamaldehyde supplementation remodels the structure of gut microbiota in early weaned rats. **(A)** Composition of gut microbiota at the phylum level analysis. **(B)** Heat map of phylum. **(C)** Relative abundance of the top four phyla. **(D)** Ratio of *Firmicutes* to *Bacteroidetes*. Data are presented as mean ± SEM. *P < 0.05 and **P < 0.01. One-way ANOVA was carried out for each comparison with HSD test.

At the phylum level, *Firmicutes* and *Bacteroidetes* were the most abundant phyla in all groups (Figures 6A,B). The relative abundance of *Firmicutes* in cinnamaldehyde groups was significantly reduced (*P* < 0.01), whereas those of *Bacteroidete, Verrucomicrobia, Proteobacteria* were increased in comparison with the control group (*P* < 0.01).

The *Firmicutes* to *Bacteroidetes* ratio (F/B ratio) has been suggested being highly correlated with many gut diseases. The increase of F/B ratio is an indicator of microbial imbalance, which is associated with intestinal diseases (29, 30). The declined F/B ratiois important for the intervention of intestinal inflammation. The ratio of F/B was 6.0 in the control group, whereas the ratios were 2.9 and 2.0 in the cinnamaldehyde groups (Figures 6C,D), respectively. These results suggested that cinnamaldehyde could attenuate the intestinal inflammatory responses via modulating the composition of gut microbiota.

**Figure 7:**
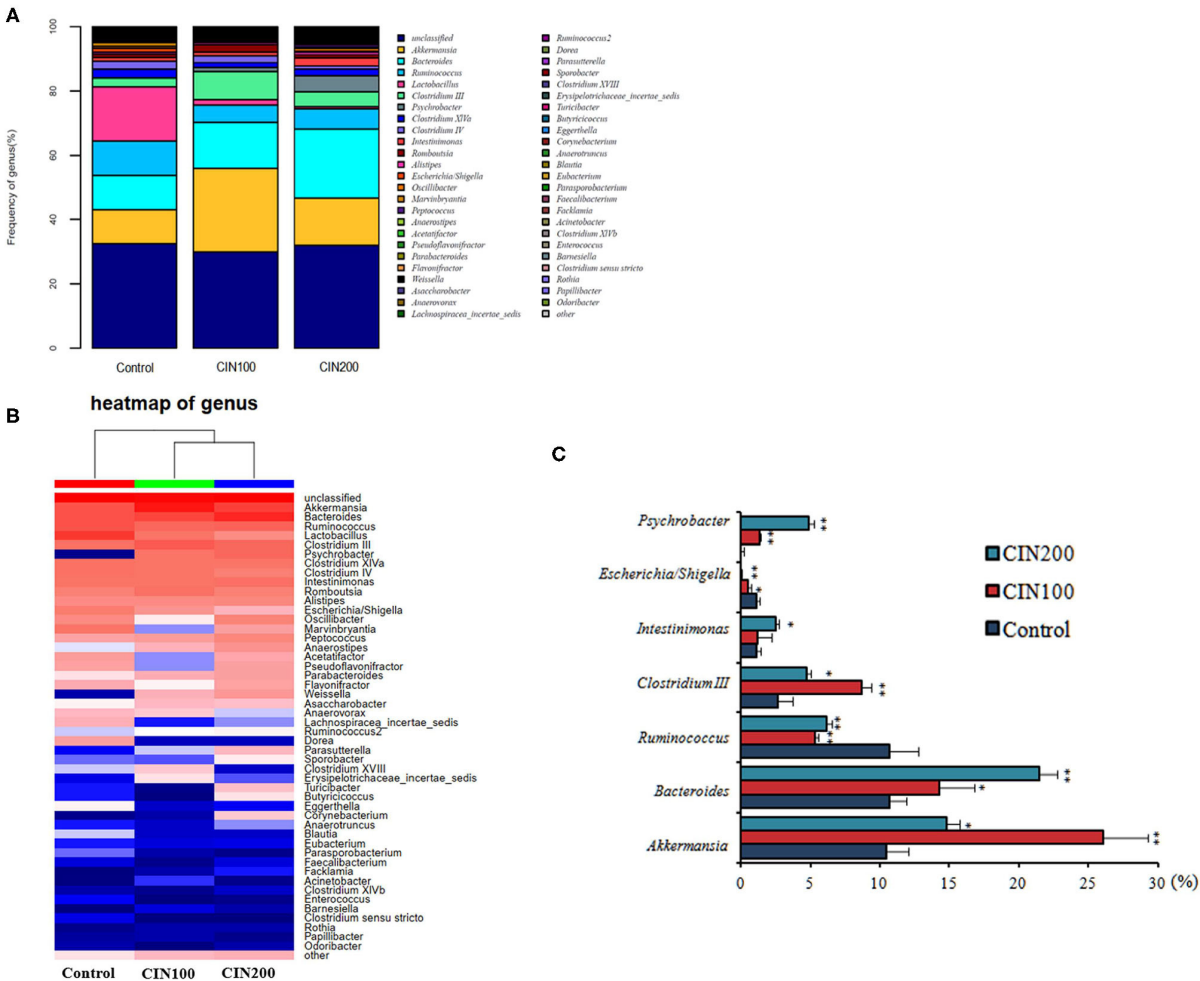
Effects of cinnamaldehyde supplementation on the abundance of intestinal bacteria at genus level. **(A)** The relative abundance of the intestinal bacteria at the genus level. **(B)** Heatmap of genus. **(C)** The relative abundances of the top seven most abundant microbial genera across all samples. *P < 0.05 and **P < 0.01. One-way ANOVA was carried out for each comparison with HSD test.

Intestinal microbiota community structure was analyzed by high-throughput sequencing of 16S rRNA. At the genus level, *Akkermansia, Bacteroides, Clostridium III, Psychrobacter, Intestinimonas* were increased, whereas those of *Ruminococcus, Escherichia/Shigella*were obviously decreased in the cinnamaldehyde treated groups (Figures 7A,B). As shown in Figure 7C, the abundance of *Akkermansia muciniphila* increased from 10.48 ± 1.77% in the control group to 26.08 ± 3.15% and 14.79 ± 2.01% in the cinnamaldehyde groups, respectively. Compared with the control group, the abundance of *Ruminococcus* was significantly decreased in CIN100 and CIN200 group. The abundance of *Escherichia/Shigella* was decreased from 1.12 ± 0.09 to 0.55 ± 0.04% and 0.09 ± 0.01%, respectively, in CIN100 and CIN200 groups. It has been reported the *A. muciniphila* is a mucin-degrading bacterium that resides in the mucus layer (31). It has been previously described that *A. muciniphila* population is reduced or depleted in inflammation (32), inflammatory bowel disease and type 2 diabetes (33, 34). The mucolytic properties seem to promote mucus renewal via a positive feedback loop which exists in a symbiotic relationship with the host (35). It has been found that *R.gnavus*can degrade colonic MUC2 (36). High abundance of *R. gnavus* is prevalent in patients with intestinal Crohn's disease and inflammatory bowel disease (37, 38). *Shigella flexneri* could colonize and invade the intestinal epithelium, resulting in severe inflammatory colitis (39). Enterotoxigenic *Escherichia coli* (ETEC) and *Shigella* are most frequently isolated pathogenic bacteria in young children with diarrhea (40). Shigella infection could induce the expression and excretion of cytokines in colonic epithelia. In this study, the expansion of intestinal beneficial bacteria and the reduction of conditional pathogenic bacteria in the cinnamaldehyde groups may be the reasons that the intestinal inflammation was attenuated by cinnamaldehyde treatment.

## Conclusion

Cinnamaldehyde, an important bioactive component extracted from cinnamonessential oils, has been found to exhibit many biological activities. To our knowledge, this is the first study to investigate the effects of cinnamaldehyde on the intestinal epithelial barrier and the gut micobiome of juvenile rats. Cinnamaldehyde treatment could promote the production of mucins in early weaned rats and upregulate the gene expression of the TJs such as ZO-1, claudin-1, and occludin. The activation of NF-κB signaling pathway was significantly suppressed and the inflammatory responses were attenuated in the colonic tissues of early weaned rats treated with cinnamaldehyde. Treatment with 100 or 200 mg/kg body weight/d of cinnamaldehyde significantly enhanced the phosphorylation of NF-κB, promoted the nuclear translocation of NF-κB, and suppressed the production of cytokines (TNF-α and IL-6). Cinnamaldehyde modulated the gut bacterial microbiota by increasing the abundance of *Bacteroidetes, Proteobacteria*, and *Verrucomicrobia* in the colon. In the cinnamaldehyde treated groups, the *Firmicutes* to *Bacteroidetes* ratio was significantly decreased. Moreover, cinnamaldehyde treatment obviously improved the diversity of colonic microbiota in the early weaned rats. These findings suggest that cinnamaldehyde should be considered as a potential drug to prevent or treat the intestinal inflammatory diseases of newborns and children. Clinical trials should be performed to confirm or refute these findings in humans.

## Data Availability Statement

The datasets presented in this study can be found in online repositories. The names of the repository/repositories and accession number(s) can be found below: NCBI BioProject, PRJNA761503.

## Ethics Statement

The animal study was reviewed and approved by the Ethical Committee in Zhejiang University.

## Author Contributions

LQ: investigation, methodology, and writing-original draft. HM: methodology and resources. TS: methodology, investigation, and data curation. XL: formal analysis, data curation, visualization, and software. JW: conceptualization, writing-review and editing, and funding acquisition. All authors contributed to the article and approved the submitted version.

## Funding

This work was funded by the National Natural Science Foundation of China (No. 31272461), the financial support from Ningbo Science and Technology Bureau Project (No.202003N4305, 2018B10095, and 2017C110017).

## Conflict of Interest

XL is employed by Ningbo Biomart Lifetech Co. Ltd. The remaining authors declare that the research was conducted in the absence of any commercial or financial relationships that could be construed as a potential conflict of interest.

## Publisher's Note

All claims expressed in this article are solely those of the authors and do not necessarily represent those of their affiliated organizations, or those of the publisher, the editors and the reviewers. Any product that may be evaluated in this article, or claim that may be made by its manufacturer, is not guaranteed or endorsed by the publisher.
