# Corrigendum: Cinnamaldehyde promotes the intestinal barrier functions and reshapes gut microbiome in early weaned rats

**DOI:** 10.3389/fnut.2022.1038451

**Published:** 2022-09-30

**Authors:** Lili Qi, Haiguang Mao, Xiaohui Lu, Tingting Shi, Jinbo Wang

**Affiliations:** ^1^School of Biological and Chemical Engineering, NingboTech University, Ningbo, China; ^2^Ningbo Biomart Lifetech Co. Ltd, Ningbo, China

**Keywords:** cinnamaldehyde, gut barrier, inflammatory responses, gut microbiota, early weaned rats

In the published article, there was an error in affiliation [1]. Instead of “[School of Biological and Chemical Engineering, Ningbo Tech University, Ningbo, China],” it should be “[School of Biological and Chemical Engineering, NingboTech University, Ningbo, China].”

The authors apologize for this error and state that this does not change the scientific conclusions of the article in any way. The original article has been updated.

## Publisher's note

All claims expressed in this article are solely those of the authors and do not necessarily represent those of their affiliated organizations, or those of the publisher, the editors and the reviewers. Any product that may be evaluated in this article, or claim that may be made by its manufacturer, is not guaranteed or endorsed by the publisher.

